# Zooming in on Early Aggression: A Cross‐Cultural and Developmental Study of Youth in the United States and Aotearoa New Zealand

**DOI:** 10.1002/ab.70043

**Published:** 2025-07-07

**Authors:** Cara S. Swit, Paula J. Fite, Seth C. Harty

**Affiliations:** ^1^ Faculty of Health University of Canterbury Christchurch Canterbury New Zealand; ^2^ Department of Clinical Child Psychology University of Kansas Lawrence Kansas USA; ^3^ Faculty of Science University of Canterbury Christchurch Canterbury New Zealand

**Keywords:** early childhood, physical aggression, proactive aggression, reactive aggression, relational aggression

## Abstract

There is ample evidence supporting developmental differences in overall rates of both physical and relational aggression. However, research evaluating developmental trends in specific acts of aggression across measures is limited, particularly in early childhood. A better understanding of what specific acts of aggression are more common in early childhood could inform assessment and identify specific behavioral targets for early prevention and intervention efforts. The current study advances extant literature by examining teacher reported rates of specific acts of aggression in samples of early childhood youth from United States and Aotearoa New Zealand. Specifically, in the U.S. sample (*N* = 322, 56.5% male), differences in rates in specific acts of physical and relational aggression (and gender differences) were compared across preschool, kindergarten, and first grade. To further evaluate specific acts of aggression in early childhood, the New Zealand sample (*N* = 200, 51.5% male) examined age differences in preschoolers (2‐, 3‐, 4‐, and 5‐year‐olds) on acts of aggression delineated by both form (physical and relational) and function (proactive and reactive) as well as gender differences. Utilizing different measures, findings indicated that while acts of aggression that require more cognitive and verbal skills occurred at high rates among older youth, overall, percentages of physical aggression were consistent across ages/grade in both early childhood samples. Boys exhibited higher percentages of physical aggression than girls, as expected. However, gender differences in relational aggression were not consistent across the samples. Data from both countries support the importance of examining specific acts of aggression.

## 1 Literature Review

While evidence suggests that forms and functions of aggression change over the course of development, there is still limited understanding of how specific acts of relational and physical aggression manifest in early childhood (e.g., Swit 2019). In this study, “specific acts” of aggression refer to discrete, observable behaviors such as hitting, kicking, excluding peers, or spreading rumors, as opposed to broad categories like physical and relational aggression. Gaining a clearer understanding of specific aggressive behaviors in preschoolers would aid in identifying targets for early intervention, ultimately aiming to prevent the escalation of aggression later on. To first establish rates of aggression, specific acts of relational and physical aggression are examined in a sample of preschool, kindergarten, and first‐grade youth from the United States (U.S) (Study 1). Recognizing the importance of understanding the functions of aggression, we then focus on reactive and proactive forms of relational and physical aggression in a sample of preschoolers (2‐ to 5‐year‐old) from Aotearoa New Zealand (NZ) (Study 2). These studies complement each other by providing an expansive view of specific aggressive behaviors in early childhood and then narrowing the focus to explore the functions of these behaviors. Both studies examine the percentages of aggression at various ages/grades, as well as potential sex differences, offering a comprehensive understanding of specific aggressive acts during early childhood development across two different cultural contexts.

### 1.1 Forms and Functions of Aggression

Aggression, defined as the intentional infliction of harm where the victim is motivated to avoid the behavior (Warburton and Anderson 2015) can take many forms and functions. During early childhood, forms of relational aggression and physical aggression receive the most empirical attention. *Relational aggression* involves the hurtful manipulation, dissolving, or threatening of peer relationships and friendships through overt behaviors such as telling another child “you can't be my friend,” and covert behaviors such as gossiping and spreading rumors about a peer (Nelson et al. 2022). In contrast, *physical aggression* is the use or threat of physical force such as punching, kicking, biting, and forcibly snatching (Nelson et al. 2022). Although relational and physical aggression are closely associated, each form of aggression follows its own distinct developmental trajectory, has different correlates, and varies in prevalence across the lifespan (Card et al. 2008; Little et al. 2003; Perry et al. 2021).

Child aggression can serve varied and distinct functions that reflect the underlying motives behind the behavior (Anderson and Bushman 2002; Dodge 1991; Ostrov et al. 2013). The primary functions of aggression are either to inflict harm or punishment on another person (i.e., reactive, hostile, impulsive, or retaliatory aggression) or to achieve a specific goal, such as intentionally planning to harm someone to obtain a desired outcome (i.e., proactive, instrumental, or planned aggression) (Little et al. 2003; Ostrov et al. 2013; Warburton and Anderson 2015). Moreover, there are item‐level and qualitative differences in aggressive behaviors (Swit 2019; Swit et al. 2024), suggesting that not only the intent behind the aggression varies but also the specific forms and functions it takes can differ significantly based on context, development, and individual differences. Similarly, different acts of aggression have varying social implications for victims, making it crucial to understand the nuances of specific aggressive behaviors to effectively address and mitigate them early in childhood.

### 1.2 Development of Aggression During the Early Years

Although extant research suggests that both relational and physical aggression are moderately stable in early childhood (e.g., Ostrov et al. 2006; Ostrov and Crick 2007), they have distinctive developmental patterns (Fite and Pederson 2018). Physical acts of aggression are evident very early on in life, with the vast majority of 6–36‐month‐old children engaging in physical aggression (Nelson et al. 2022). Overall, data suggest that physical aggression peaks between 2 and 3 years of age and continues to decrease from the preschool years throughout childhood for most children (Fite and Pederson 2018; Nelson et al. 2022). Note, however, that physical aggression may also become more planful and/or covert as well as more verbal (e.g., threaten harm) in nature as cognitive skills and behavioral regulation improve (Fite and Pederson 2018; Swit 2019). That is, rates of specific acts of physical aggression likely change as youth age.

Relational aggression, on the other hand, typically emerges a bit later, with relational acts of aggression observed as early as 30 months (Fite and Pederson 2018; Nelson et al. 2022). Rates of relational aggression tend to increase as children age and enter school (Fite and Pederson 2018). Importantly, there is some limited evidence suggesting that relational aggression may look different at various stages of development. As children age and their cognitive and verbal skills improve, their methods of damaging social relationships become more sophisticated (Fite and Pederson 2018). Specifically, within early childhood, acts of relational aggression appear to be more direct (rather than indirect) in nature and immediate rather than manifest as future acts of retaliation (Casas and Bower 2018; Swit 2019). For example, in a sample of children attending preschool, both educators and parents reported that acts such as “telling a child they won't play with them anymore” were more commonly observed than acts such as “getting others to dislike a peer” (Swit 2019). However, research examining the rates of specific aggressive behaviors in early childhood is limited, highlighting the need for further investigation to identify which acts should be assessed and targeted for early prevention and intervention (Swit 2019; Swit and Slater 2021). This approach can reveal patterns in children's aggressive behaviors that may be obscured when only considering aggression categories.

With regard to the functions of aggression, only limited research exists examining the developmental trajectories of proactive and reactive aggression among preschoolers (Fite et al. 2023). Extant findings are mixed (e.g., Murray‐Close and Ostrov 2009), but there is some evidence suggesting that both proactive and reactive aggression are stable as early as 30 months old (Ostrov and Crick 2007) and continue to remain stable throughout early childhood (Evans et al. 2019). However, more research, particularly examining how common specific acts of proactive and reactive aggression are in early childhood, is warranted.

### 1.3 Gender Differences in Aggression

There is substantial evidence indicating that boys are more physically aggressive than girls throughout the lifespan, with these effects first emerging in early childhood (Fite and Pederson 2018; Nelson et al. 2022). In contrast, gender differences in relational aggression are complex, with mixed findings in existing research (for review see, Fite and Pederson 2018; Nelson et al. 2022). While some studies suggest similar rates between boys and girls (Morine et al. 2011; Stauffacher and DeHart 2005), others indicate higher rates among girls (Fite and Pederson 2018; Nelson et al. 2022). There is limited research examining gender differences in proactive and reactive aggression in early childhood (Fite et al. 2023). Some evidence suggests that girls may be less likely to engage in proactive and reactive aggression than boys (Evans et al. 2019); however, findings are mixed (Murray‐Close and Ostrov 2009).

Moreover, gender differences may depend on the specific acts of aggression exhibited. For example, Swit (2019) asked educators and parents of early childhood age youth to report on acts of aggression they observed and intervened in over the past month and whether these behaviors were more common among boys or girls (or both). While parents and caregivers rated relationally aggressive acts as more likely to occur among girls and physically aggressively acts as more likely to occur among boys, there were certain physical (i.e., “hurting others by pinching them” and “ruining other peer's things”) and relational items (i.e., “stopping a peer from being the play group” and “verbally threatening to keep a peer out of a playgroup”) that educators indicated both boys and girls equally engaged in (Swit 2019). Thus, more research examining gender differences in rates of specific acts of aggression is needed.

### 1.4 Cross‐Cultural Differences in Relational and Physical Aggression

While aggression exhibits both universal and culture‐specific dimensions (Severance et al. 2013), most studies examining physical and relational aggression in children have been conducted in Western countries, particularly the United States (Severance et al. 2013). Lansford et al. (2012) examined childhood relational and physical aggression from 1410 families across nine countries. Countries were notable for a mixture of individualistic and collectivist cultures and existed across a continuum of social norm expectancies. Across all countries, moderate associations were found between relational and physical aggression, such that more frequent relational aggression was associated with more frequent physical aggression. However, countries differed in mean levels of child‐reported aggression, a finding which the authors hypothesized was likely due to cultural differences surrounding normative beliefs about aggression. Culturally mediated differences in aggression are unlikely to be solely attributed to East/West, individualistic/collectivist, or high/low‐income distinctions. This is evidenced by studies that have found similarities in physical and relational aggression among Chinese children in Hong Kong (Tseng et al. 2013) and mainland China (Nelson et al. 2006) compared to Western children. This study extends previous research by specifically examining whether differences in specific acts of aggression differ across cultural contexts in the U.S. and NZ.

Additionally, various measures are used to assess aggression (Swit and Slater 2021); however, few studies have examined the rates of different aggressive acts. Instead, researchers have typically computed total scores for relational and physical aggression, making it difficult to examine and compare specific aggressive behaviors within and across countries. An exception of this trend comes from the Organisation for Economic Co‐operation and Development (Organisation for Economic Co‐operation and Development, OECD 2023) Programme for International Student Assessment (PISA), which reports descriptive data on specific acts of aggression among 15 year old participants. It was found that 9.1% of young people in NZ and 5.2% in the U.S. reported that other students made fun of them at least once a week. Following this, 3.6% (NZ) and 3% (U.S.) said they were purposely excluded by others weekly, while 2.7% (NZ) and 2.5% (U.S.) experienced rumors being spread about them. Physical aggression was less frequent, with 2.5% (NZ) and 1.2% (U.S.) reporting being hit or pushed around weekly, and 2.4% (NZ) and 1.5% (U.S.) threatened by other students. Additionally, 1.6% (NZ) and 0.8% (U.S.) had their belongings taken or destroyed, while 0.9% (NZ) and 0.7% (U.S.) were involved in a physical fight. The least reported behavior was being forced to give money to peers, experienced by 1.2% (NZ) and 0.7% (U.S.) of students. It is important to note that these percentages are descriptive and do not indicate whether the differences between countries are statistically significant. Nonetheless, these rates of specific aggressive behaviors, which indicate both similarities and differences in how youth in the U.S. and NZ exhibit aggression, provide a level of granularity not possible in the comparison of collapsed item means. Understanding similar patterns in early childhood is critical, as specific aggressive behaviors during this developmental period may serve as precursors to more serious aggression and violence in adolescence and adulthood. Examining how aggressive behaviors develop across different cultural contexts can also provide insight into the early socialization of aggression.

### 1.5 School Context in the United States and Aotearoa New Zealand

Understanding the development of aggression in early childhood requires examination of the social and environmental contexts in which children participate. From an ecological systems perspective (Bronfenbrenner 1979), early childhood education (ECE) settings are a critical part of children's mesosystem, supporting their opportunities for social learning, peer interaction, and behavioral regulation. Although both the U.S. and NZ provide ECE as a critical foundational context for children's development, key differences in the structure and approach the two countries take may influence children's socialization processes and, in turn, their aggressive behaviors. While both countries are considered individualistic societies, each has its own distinct cultural context that shapes expectations and expressions of aggression. For instance, New Zealand's bicultural educational approach and emphasis on play‐based learning contrast with the more structured and academically focused environments common in the U.S. These cultural distinctions and expectations may influence the types and social acceptability of aggressive behaviors observed in each country.

Within the U.S., ECE is highly heterogeneous, with services provided through a combination of public, private, and home‐based settings. Children begin attending childcare from infancy, and it is common for infants and toddlers to be placed in care from a very young age due to parental work demands and limited parental leave policies (Brooks–Gunn et al. 2002). Preschool programs typically begin at approximately 3 years of age and can be either part‐time (half day; 2–3 days/week) or full‐time. These programs can have specific age or mixed age classrooms. For most states, youth must turn 5 years of age within one to 2 months of the academic year commencing to attend kindergarten, and states differ on whether these youth attend half or full day kindergarten (Education Commission of the States 2018). All youth attend full‐day school by first grade.

In contrast, NZ offers a more diverse range of early learning services, including both teacher‐led and parent‐led options. Teacher‐led options include Kindergartens, which typically offer part‐day education (around 5 h) for children aged 2–5 years in one mixed‐aged classroom, and ECE centres, which offer full‐day care (up to 11 h) for children from birth to 5 years, often organized into age‐specific classrooms such as nursery (0–2 years), toddler (2–3.5 years), preschool (3.5–5 years). ECE centres are the most common service type attended by children in NZ (Education Counts 2024a). Additionally, NZ has parent‐led services such as playcentres, where parents are actively involved in the management and operation of the service, and Kōhanga reo offer ECE in a Māori language and cultural context, playing a crucial role in the revitalization of te ao Māori. As of 2023, 95.6% of NZ children aged 4 regularly attended an early learning services in the 6 months before starting school (Education Counts 2024b). In contrast, approximately 59% of 3–5‐year‐olds in the U.S. were enrolled in preschool or kindergarten programs in 2022 (National Center for Education Statistics 2024). Unlike in the U.S., NZ children typically start full‐day school on or shortly after their fifth birthday, though attendance is not compulsory until age 6. New Zealand's bicultural educational philosophy and emphasis on play‐based, child‐led early learning environments may influence children's socialization and engagement in aggression differently than more structured and academically focused early childhood environments common in the U.S. Observational research demonstrates that children in mixed‐age classrooms engage in longer, more frequent, and more positive peer interactions, and fewer negative interactions than those in same‐age classrooms, supporting the development of social skills that can help prevent aggression (Wu et al. 2021). Play‐based early childhood environments also provide opportunities for socio‐dramatic play, peer negotiation, and problem‐solving, thereby promoting young children's self‐regulation and prosocial behavior (Ginsburg 2007; Miller and Almon 2009). In contrast, academically focused classrooms typical of those in the U.S. may limit these opportunities, potentially leading to increased stress and aggression in children (Miller and Almon 2009). Taken together, these findings suggest that the structural and philosophical differences between New Zealand and the U.S. are likely to shape child aggression. By examining specific acts of aggression in samples of children from both countries, this study will contribute to our understanding of specific acts of aggression that may be universal or culturally specific.

### 1.6 The Current Study

Although the forms and functions of aggression during early childhood have been studied, much of the previous research has focused on broad constructs rather than specific behaviors. By focusing on item‐level data, this study addresses this gap to clarify how behaviors compare across measures and countries (Swit 2019). This focus on specific acts would provide helpful information to inform developmental and culturally sensitive interventions for preschool age youth. The present studies contribute to our understanding of relational and physical aggression, as well as reactive and proactive aggression, by examining the percentage of youth who engage in specific acts of relational and physical aggression among 2‐ to 7‐year‐olds and evaluating gender differences. Additionally, these studies provide observational cross‐cultural insights into aggression rates in preschool age youth by including two separate samples, one from the United States and another from Aotearoa New Zealand. While this study does not aim to provide nationally representative estimates of early childhood aggression rates in the U.S. or NZ, its focus on the prevalence of specific aggressive acts within geographically distinct samples allows us to descriptively identify patterns of these aggressive behaviors across early childhood development within two distinct cultural contexts. The aims of the current paper were (a) to examine and descriptively compare the percentages of specific acts of teacher‐reported relational and physical aggression utilizing different measures in two culturally diverse countries—the U.S. and NZ, and (b) to explore age (Study 1: 3–7 year olds, Study 2: 2–5 year olds) and gender differences in child relational and physical aggression across these samples. Additionally, Study 2 investigates specific acts of relational and physical aggression according to reactive and proactive functions of behavior. This expanded approach was possible because of the available teacher‐reported data for Study 2, which included distinctions between relational and physical aggression and reactive and proactive aggression. Examining both forms and functions of aggression offers a nuanced examination of the motivations underlying specific acts of aggression used by young children. Based on the literature reviewed (e.g., Nelson et al. 2022), we predicted physical aggression and reactive functions of aggression to be more common in younger children and relational aggression and proactive functions of aggression to be more common in older children. While older NZ youth exhibit higher rates of specific acts of aggression than U.S. peers, play‐based learning, which is central to NZ's early childhood curriculum, is associated with *reduced* aggression in young children (Ginsburg 2007; Miller and Almon 2009). Our hypothesis that NZ preschoolers will show lower aggression is exploratory, given the lack of data on specific acts of aggression and potential cultural differences in early child aggression. We expected boys to have higher rates of physical aggression (Fite and Pederson 2018; Nelson et al. 2022). Given the mixed gender findings for relational aggression, we predicted minimal to no gender differences in teacher reports of relational aggression (Swit and Slater 2021).

## 2 Methods

### 2.1 Study One

#### 2.1.1 Participants

Data included teacher reports of youth from a Midwestern city in the U.S. in which one public elementary school serves the entire community. Preschool youth attended half day, and kindergarten and first grade students attended full‐day school. Teacher participation was high, with 90% (18 out of 20) of preschool through first‐grade teachers completing student surveys. Note that 17 teachers completed surveys for all students in their classroom, while one teacher only partially completed the student surveys. This resulted in data being available for 322 youth (56.5% male). Youth ranged from 3 to 7 years of age, with 28.9% in preschool (*N* = 93; 54.8% male), 25.5% in kindergarten (*N* = 82; female; 56% male); and 45.6% in first grade (*N* = 147; 57.8% boys).

Within the school district, 85% of students were non‐Latinx White, 5% Black, 5% Latinx, 2% Asian, 2% Native American, and 1% multiracial (National Center for Education Statistics 2019). Just over one in three students (36%) received free or reduced lunch (National Center for Education Statistics 2019). The age, gender, ethnicity, and professional experience of teachers was not available for this study.

#### 2.1.2 Measures

##### 2.1.2.1 Demographics

The school provided information on grade level and classroom placements for surveys to be distributed. Gender was reported on by teachers, selecting boy (0) or girl (1).

##### 2.1.2.2 Aggression

Physical and relational aggression were assessed using items developed by Crick and colleagues (Crick 1996; Crick and Bigbee 1998; Crick et al. 1997). Specifically, teachers reported on three items to assess relational aggression and three items to assess physical aggression (see Table 1). Teachers responded to the items using a 5‐point Likert scale (1 = Never to 5 = Almost Always). Teacher reports of these items have been found to be valid and reliable among preschool age youth (Crick et al. 1997).

**TABLE 1:** Specific aggressive items used in Study 1 (United States) and Study 2 (New Zealand).

	Relational aggression	Physical aggression
Study 1 (United States)	When mad, gets even by keeping the person from being in their group of friends	Hits, kicks, punches others
	When mad at a person, ignores or stops talking to them	Pushes and shoves others
	Tries to make other kids not like a certain person by spreading rumors about them	Tells other kids beat them up unless to what they say
	**Reactive**	
Study 2 (New Zealand)	If other children hurt this child, s/he often keeps them from being in their group of friends (2)	When this child is hurt by someone, s/he will often physically fight back (1)
	When s/he is angry at others, this child will often tell them that s/he won't be their friend anymore (9)	If other children make this child mad, s/he will often physically hurt them (3)
	When s/he is upset with others, this child will often ignore or stop talking to them (10)	If other children anger this child, s/he will often hit, kick, or punch them (8)
	**Proactive**	
	To get what this child wants, s/he often tells others that s/he won't be their friend anymore (11)	This child often starts physical fights to get what s/he wants (5)
	This child often says “you can't come to my birthday party” to other children to get what s/he wants (13)	This child often threatens others physically to get what s/he wants (6)
	To get what this child wants, s/he often will ignore or stop talking to others (14)	This child often hits, kicks, or punches to get what s/he wants (12)

#### 2.1.3 Procedure

Study procedures were approved by the author's institutional review board and the school administrators before the study commencing. Teachers were recruited during a teacher in service meeting. All teachers were provided with a brief overview of the study and those teachers who agreed to participate provided written consent. Teachers then completed an online survey for each student. Each student survey took no more than 10 min to complete. Teachers were provided with $50 if they completed all student surveys and $25 if they completed surveys for some (but not all) students.

### 2.2 Study Two

#### 2.2.1 Participants

Two hundred children (51.5% males) from five community‐based kindergartens in the South Island of New Zealand participated in this study. Children were aged between 25 months and 71 months (*M* age = 46.13 months, SD = 9.81 months), with 15.5% (*N* = 31) 2‐year‐olds, 39% (*N* = 78) 3‐year‐olds, 38% (*N* = 76) 4‐year‐olds, and 7.5% (*N* = 15) 5‐year‐olds. The kindergartens were in communities representing diverse socioeconomic status. Participation rates at all kindergartens exceeded 80%. The ethnic composition of the sample was 41.5% NZ European, 8.5% Māori, 8.5% Asian, and 5.5% Pacific Islander.

Eighteen kindergarten teachers (87.5% female, *M* age = 57.70 years, SD = 3.61 years) with 9–45 years teaching experience (*M* = 23.3 years, SD = 7.40) completed behavioral reports of children's aggressive behavior. Sixteen teachers identified as Caucasian. Twelve teachers had completed a Bachelor's degree; six had completed a Diploma.

#### 2.2.2 Measures

##### 2.2.2.1 Demographics

Child age (in months) and gender was reported on by teachers, selecting boy (0) or girl (1).

##### 2.2.2.2 Teacher Report of Forms and Functions of Aggression

The forms and functions of children's aggression were measured using the Preschool Proactive and Reactive Aggression Teacher Report (PPRA‐TR) (Ostrov and Crick 2007). Using a 5‐point Likert‐type scale (0 = never or almost never true; 4 = always or almost always true), teachers rated the frequency of children's aggressive behaviors on four subscales: reactive‐relational aggression (R‐RA), proactive‐relational aggression (P‐RA), reactive‐physical aggression (R‐PA), and proactive‐physical aggression (P‐PA). See Table 1 for a list of items. Teacher reports of these items have been found to be valid and reliable among preschool age youth (Ostrov and Crick 2007).

#### 2.2.3 Procedure

This study was approved by the author's institutional ethics review committee [approval number 2020/04/ERHEC]. Written informed consent was obtained from parents and teachers before teachers completed the child behavioral reports. Teachers who had known the child for at least eight weeks completed the behavioral reports. These behavioral reports were part of a larger survey packet that teachers completed for each participating child. Each packet of surveys took teachers approximately 15 min to complete.

### 2.3 Data Analysis

In Study One, child aggression scores were collected from eighteen teachers within a single school. Each teacher reported on between six and twenty‐five children. In Study Two, child aggression scores were collected from teachers across five kindergartens. Three kindergartens had three teacher informants, and two kindergartens had four teacher informants. Each teacher reported on between three and eighteen children.

To assess the degree of interdependence within the school/kindergarten, intraclass correlation coefficients (ICC) were calculated for each aggression item to assess whether there was a clustering effect at the teacher (Study 1 and Study 2) and kindergarten (Study 2) level. Results showed that there was no significant variance within or between groups for physical aggression. However, some variance was found for relational aggression, particularly at the teacher level (see Supplementary Tables 1, 2). Given the small number of teachers and schools/kindergartens in each study, the power to detect clustering effects may be limited. Multilevel modeling typically requires a minimum of 20 groups (Lai and Kwok 2014), suggesting that the sample size in both studies may be underpowered to fully assess clustering effects. Therefore, the results of this study should be interpreted with this limitation in mind.

We used well established and reliable measures of early childhood aggression (Swit and Slater 2021) in both Study 1 and Study 2. Although the measures differed in their structure and focus on aggression—Study 1 capturing broad aggressive behaviors and Study 2 differentiating between forms and functions of aggression—they were conceptually aligned. Specifically, comparable aggressive behaviors across the two measures are presented in Table 1.

First, teacher reports of youth aggression were dichotomized by giving a score of 0 (never), or 1 (any other response) for each aggression item. Grade and age level differences in percentages of youth who engaged in each act of aggression were examined using binary logistic regressions, with each dichotomous aggression item regressed on grade or age as a categorical independent variable (Ranganathan et al. 2017). Given the interest in preschool age youth, preschool was utilized as the referent group in U.S. sample analyses. For the NZ sample, age 4 was used as the referent group given that this was the approximate mean age of the sample. However, note that we also ran analyses with age 5 as the referent group, and the pattern of findings was consistent. Odds ratios (OR) were used to examine the effect size of each grade or age level comparison. Next, Chi‐square analyses were examined to determine differences between boys and girls.^1^ The Phi (φ) statistic was used to measure the effect size between child gender and each aggressive act, where values of 0.1, 0.3, and 0.5 are considered small, medium, and large effects, respectively (Cohen 1988). Note that there were no missing data associated with the aggression items, therefore, no missing data approach was needed.

## 3 Results

### 3.1 Aggressive Acts Exhibited

The findings from Study 1 and Study 2 reveal several patterns in the percentages of aggressive acts. Across all grade levels (preschool, kindergarten, and first grade), the most common aggressive act was ignoring peers when mad at them (32%–42% of the sample), followed by pushing and shoving others (28‐39% of the sample). Hitting others (22%–28% of the sample) and peer exclusion (13%–16% of the sample) were the next most common aggressive acts reported by teachers in preschool and kindergarten. In contrast, in the first grade, peer exclusion (approximately 35% of the sample) and spreading rumors (approximately 31% of the sample) were more common. The least common aggressive acts were verbal aggression, such as telling others to beat up someone unless they comply, with prevalence ranging from 2.2% in preschool to 13.6% in first grade.

When examining forms and functions of aggressive acts in children aged 2–5 years in Study 2, reactive aggression was most commonly reported. The most common forms of aggression included keeping peers out of their friendship group when hurt (61.5% of the sample) and ignoring peers when upset with them (60.5% of the sample). Physically fighting back when hurt by someone and telling their peers they won't be their friend anymore, when angry at others were present in over half of the sample (54%). The least common aggressive act involved threatening others physically to gain compliance, with 10.5% of the sample engaging in this behavior.

### 3.2 Grade Level and Age Differences in Aggressive Acts

**FIGURE 1:**
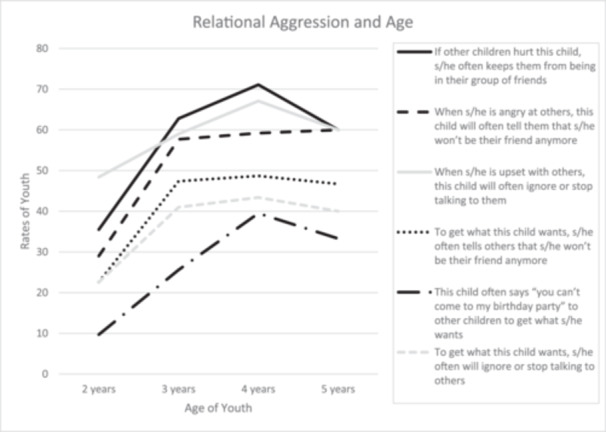
Grade‐level differences in specific acts of relational and physical aggression for Study 1 (United States). *Note:* Black lines indicate statistically significant effects (*p* < 0.05), such more 1st grade (not kindergarten) youth engage in this act of aggression than preschool youth.

As depicted in Figure 1, binary logistic regressions indicated significant grade level differences in relational and physical aggression between preschoolers and first graders in Study 1. First graders were 7.17 times more likely than preschoolers to *threaten to beat up other children* (*p* < 0.01), 2.76 times more likely to *keep peers from being in their group of friends* (*p* < 0.01), and 4.69 times more likely to *spread rumors* (*p* ≤ 0.01) (see Supporting Information S1: Table 3). No other differences were found between preschoolers and first graders (*p*s > 0.83), nor were there any significant grade level differences found between preschoolers and kindergarteners (*p*s < 0.16) (see Supporting Information S1: Table 3).

**FIGURE 2:**
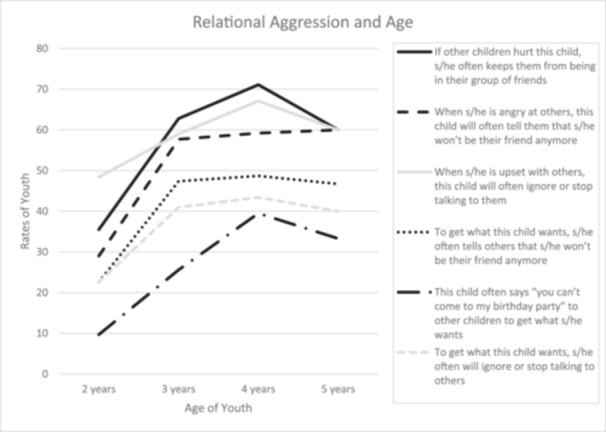
Age differences for reactive and proactive relational aggression for Study 2 (New Zealand). *Note:* Black lines indicate statistically significant age differences (*p* < 0.05).

Findings from Study 2 further refined patterns seen in Study 1 by examining specific age level differences in relational and physical forms, and reactive and proactive functions of aggression. As shown in Figure 2, binary logistic regression revealed significant age differences in four relationally aggressive acts. Statistically significant differences were found for two reactive relational aggression behaviors: “*If other children hurt this child, s/he often keeps them from being in their group of friends”* (*p* = 0.01) and *“When s/he is angry at others, this child will often tell them that s/he won't be their friend anymore”* (*p* = 0.02), and two proactive relational aggressive behaviors: *“To get what this child wants, s/he often tells others that s/he won't be their friend anymore”* (*p* = 0.02), and *“This child often says, “you can't come to my birthday party” to other children to get what s/he wants”* (*p* = 0.01).

**FIGURE 3:**
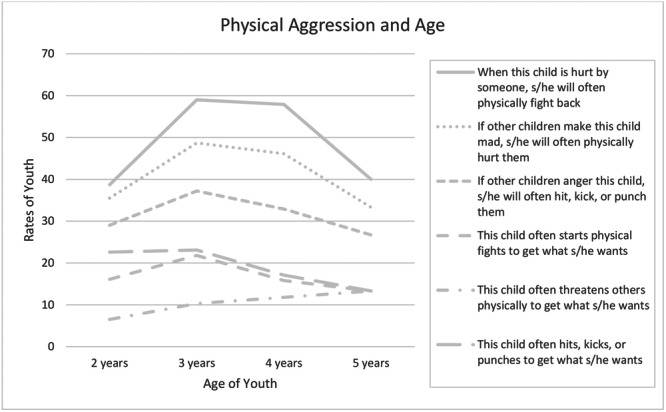
Age differences for reactive and proactive physical aggression for Study 2 (New Zealand).

Two‐year‐olds were significantly less likely than 4‐year‐olds to engage in these behaviors. Specifically, they were 78% less likely to retaliate by excluding others (*p* < 0.001), 72% less likely to tell others they won't be friends anymore when angry (*p* = 0.01), 69% less likely to use the threat of ending a friendship to get what they want (*p* = 0.02), and 84% less likely to use the threat of exclusion from a birthday party to get what they want (*p* = 0.01). In contrast, 3‐year‐olds (*ps* > 0.07) and 5‐year‐olds (*ps* > 0.40) did not differ significantly from 4‐year‐olds in these behaviors (see Supporting Information S1: Table 4). No significant age differences were found for reactive and proactive physical aggression (see Figure 3 and Supporting Information S1: Table 5).

### 3.3 Gender Differences in Aggressive Acts

**FIGURE 4:**
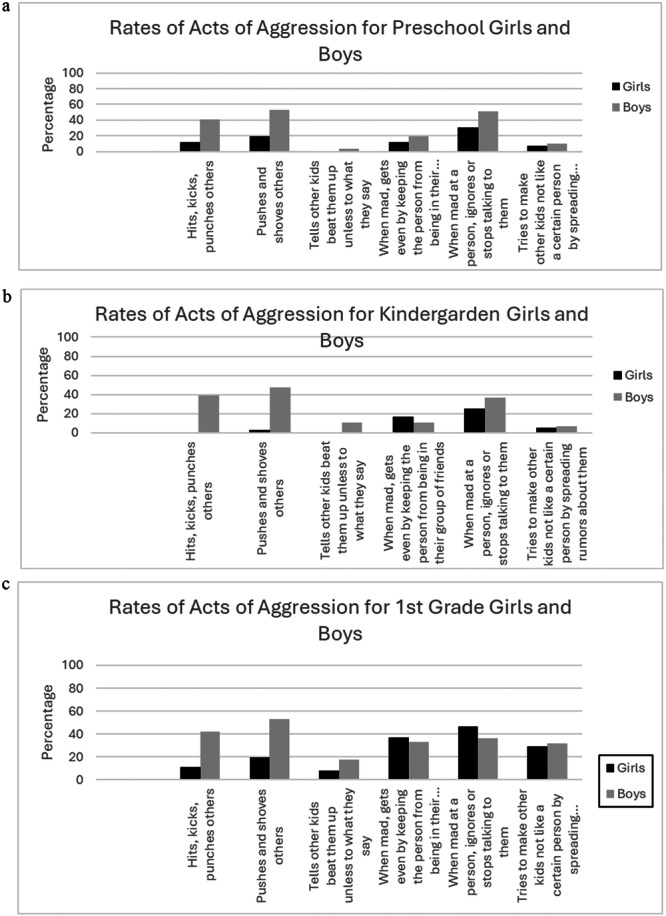
(a) Rates of specific acts of aggression for preschool girls and boys for Study 1 (United States). (b) Rates of specific acts of aggression for kindergarten girls and boys for Study 1 (United States). (c) Rates of specific acts of aggression for first‐grade girls and boys for Study 1 (United States).

Chi‐square analyses indicated small to moderate gender differences in physical aggression across all three grade levels in Study 1. Boys were more likely than girls to engage in higher rates of the *hitting, kicking, and punching* item as well as the *pushing and shoving* (see Tables 2, 3, 4, and Figure 4). Additionally, in kindergarten more boys than girls *threatened to beat others up* (see Table 3, Figure 4b). However, only one significant gender difference in relational aggression was found, such that in preschool more boys engaged in *ignoring or stop talking* than girls (see Table 2, Figure 4a).

**TABLE 2:** Frequency count of teacher‐reported aggression in preschool youth, for the total sample and by gender for Study 1 (United States).

Item	Boys and girls	Girls	Boys	*Χ* ^2^
Hits, kicks, punches others	28%	11.9%	41.2%	*Χ* ^2^ = 9.80, ***p* < 0.001,** φ = 0.33
Pushes and shoves others	37.6%	19%	52.9%	*Χ* ^2^ = 11.27, ***p* < 0.001,** φ = 0.35
Tells other kids beat them up unless to what they say	2.2%	0%	3.9%	*Χ* ^2^ = 1.68, *p* = 0.19, φ = 0.14
When mad, gets even by keeping the person from being in their group of friends	16.1%	11.9%	19.6%	*Χ* ^2^ = 1.01, *p* = 0.32, φ = 0.10
When mad at a person, ignores or stops talking to them	41.9%	31.0%	51.0%	*Χ* ^2^ = 3.80, ***p* = 0.05,** φ = 0.20
Tries to make other kids not like a certain person by spreading rumors about them	8.6%	7.1%	9.8%	*Χ* ^2^ = 0.21, *p* = 0.65, φ = 0.05

*Note: N* = 93, 54.8% male, bolded values indicate *p* < 0.05.

**TABLE 3:** Frequency count of teacher‐reported aggression in kindergarten youth, for the total sample and by gender for Study 1 (United States).

Item	Boys and girls	Girls	Boys	*Χ* ^2^
Hits, kicks, punches others	22%	0%	39.1%	*Χ* ^2^ = 18.05, ***p* < 0.001,** φ = 0.47
Pushes and shoves others	28%	2.8%	47.8%	*Χ* ^2^ = 20.31, ***p* < 0.001,** φ = 0.50
Tells other kids beat them up unless to what they say	6.1%	0%	10.9%	*Χ* ^2^ = 4.17, ***p*** = ** 0.04,** φ = 0.23
When mad, gets even by keeping the person from being in their group of friends	13.4%	16.7%	10.9%	*Χ* ^2^ = 0.58, *p* = 0.45, φ = 0.08
When mad at a person, ignores or stops talking to them	31.7%	25%	37%	*Χ* ^2^ = 1.33, *p* = 0.15, φ = 0.13
Tries to make other kids not like a certain person by spreading rumors about them	6.1%	5.6%	6.5%	*Χ* ^2^ = 0.03, *p * = 0.86, φ = 0.02

*Note: N* = 82, 56% male, bolded values indicate *p* < 0.05.

**TABLE 4:** Frequency count of teacher‐reported aggression in first grade youth, for the total sample and by gender for Study 1 (United States).

Item	Boys and girls	Girls	Boys	*Χ* ^2^
Hits, kicks, punches others	29.3%	11.3%	42.4%	*Χ* ^2^ = 16.72, ***p* < 0.001,** φ = 0.34
Pushes and shoves others	38.8%	19.4%	52.9%	*Χ* ^2^ = 17.04, ***p* < 0.001,** φ = 0.34
Tells other kids beat them up unless to what they say	13.6%	8.1%	17.6%	*Χ* ^2^ = 2.80, *p* = 0.09, φ = 0.14
When mad, gets even by keeping the person from being in their group of friends	34.7%	37.1%	32.9%	*Χ* ^2^ = 0.27, *p *= 0.60, φ = 0.04
When mad at a person, ignores or stops talking to them	40.8%	46.8%	36.5%	*Χ* ^2^ = 1.58, *p* = 0.21, φ = 0.10
Tries to make other kids not like a certain person by spreading rumors about them	30.6%	29%	31.8%	*Χ* ^2^ = 0.13, *p *= 0.73, φ = 0.03

*Note: N* = 147, 57.8% boys, bolded values indicate *p* < 0.05.

Chi‐square analyses for Study 2 revealed that boys were more likely than girls to engage in the reactive physical aggression such as “*When this child is hurt by someone, s/he will often physically fight back*” and “*If other children anger this child, s/he will often hit, kick, or punch them*.” Boys were also more likely to use proactive physical aggression, such as “*This child often hits, kicks, or punches to get what s/he wants”* (see Table 5), and these effects were small in nature.

**TABLE 5:** Frequency count of teacher‐reported physical aggression, separated by function and by gender for Study 2 (New Zealand).

	Boys and girls	Girls	Boys	*Χ* ^2^
Reactive				
When this child is hurt by someone, s/he will often physically fight back (1)	54%	46.4%	61.2%	*Χ* ^2^ = 4.39, ***p*** = **0.04**, φ = 0.15
If other children make this child mad, s/he will often physically hurt them (3)	44.5%	38.1%	50.1%	*Χ* ^2^ = 3.08, *p* = 0.08, φ = 0.12
If other children anger this child, s/he will often hit, kick, or punch them (8)	33.5%	25.8%	40.8%	*Χ* ^2^ = 5.05, ***p*** = **0.03**, φ = 0.16
Proactive				
This child often starts physical fights to get what s/he wants (5)	18%	13.4%	22.3%	*Χ* ^2^ = 2.70, *p* = 0.10, φ = 0.12
This child often threatens others physically to get what s/he wants (6)	10.5%	6.2%	14.6%	*Χ* ^2^ = 3.73, *p* = 0.05, φ = 0.14
This child often hits, kicks, or punches to get what s/he wants (12)	20%	12.4%	27.2%	*Χ* ^2^ = 6.85, ***p*** = **0.01**, φ = 0.1

*Note:* Bolded values indicate p < 0.05.

In contrast, a different pattern of gender differences was identified for relational aggression. Teachers reported that girls were more likely than boys to engage in reactive relational aggression, such as *“When s/he is angry at others, this child will often tell them that s/he won't be their friend anymore.”* Additionally, girls were more likely than boys to use proactive relational aggression behaviors such as *“To get what this child wants, s/he often tells others that s/he won't be their friend anymore”* and *“This child often says, “you can't come to my birthday party” to other children to get what s/he wants”* (see Table 6), and these effects were small in nature.

**TABLE 6:** Frequency count of teacher‐reported relational aggression, separated by function for Study 2 (New Zealand).

	Boys and girls	Girls	Boys	*Χ* ^2^
Reactive				
If other children hurt this child, s/he often keeps them from being in their group of friends (2)	61.5%	67%	56.3%	*Χ* ^2^ = 2.42, *p* = 0.12, φ = 0.11
When s/he is angry at others, this child will often tell them that s/he won't be their friend anymore (9)	54%	63.9%	44.7%	*Χ* ^2^ = 7.46, **p** = **.01**, φ = 0.19
When s/he is upset with others, this child will often ignore or stop talking to them (10)	60.5%	67%	54.4%	*Χ* ^2^ = 3.34, *p* = 0.07, φ = 0.13
Proactive				
To get what this child wants, s/he often tells others that s/he won't be their friend anymore (11)	44%	52.6%	35.9%	*Χ* ^2^ = 5.62, **p** = **.02**, φ = 0.17
This child often says “you can't come to my birthday party” to other children to get what s/he wants (13)	29%	38.1%	20.4%	*Χ* ^2^ = 7.65, **p** = **.01**, φ = 0.20
To get what this child wants, s/he often will ignore or stop talking to others (14)	39%	44.3%	34%	*Χ* ^2^ = 2.25, *p* = 0.13, φ = 0.11

*Note:* Bolded values indicate p < 0.05.

## 4 Discussion

While much is known about the trajectories and consequences of aggression in children, the manifestation of specific acts of aggression in very young children remains relatively underexplored (Swit 2019). This study examined teacher‐reported percentages of youth who engaged in specific aggressive behaviors. Specifically, Study 1 analyzed trends in specific acts of relational and physical aggression among U.S. children from preschool through first grade, while Study 2 analyzed the functions of these relationally and physically aggressive acts in a preschool sample from NZ.

### 4.1 Comparisons Across U.S. and NZ Youth

A key strength of this study is that despite using two different measures of aggression across Study 1 and Study 2, comparable descriptive patterns emerged in the findings, as well as some differences. In both countries, preschoolers exhibited comparable percentages of physical aggression, specifically, hitting, kicking, and punching. Boys in both the U.S. and NZ were reported to engage in these specific acts of aggression more commonly than girls. Although the percentages of specific acts of physical aggression were higher than those reported in studies involving 15‐year‐olds in each country (Organisation for Economic Co‐operation and Development, OECD 2023), the consistent trend across age groups suggest that, from an early age, children in both the U.S. and NZ engage in hitting, kicking, and punching at similar rates. In contrast, the specific act of directing others to beat up a peer was rated as the least common act of physical aggression in both countries.

When examining relational aggression, ignoring or not talking to peers was commonly reported by teachers in both countries. Notably, boys in the U.S. were rated higher on this behavior, whereas girls in NZ engaged in this behavior more often. In Study 2, girls were also more likely than boys to engage in other relationally aggressive behaviors, suggesting that relational aggression may be more gender‐differentiated in NZ compared to the U.S. Further, the lack of differences in relationally aggressive acts between preschoolers and kindergarteners in Study 1 aligns with Study 2's finding that 3‐year‐olds did not differ significantly from 4‐year‐olds in their aggression, suggesting a possible developmental threshold at approximately age four when relational aggression becomes more prominent. This alignment of results across different aggression measures in each study strengthens the robustness of these findings, suggesting that these observed patterns reflect meaningful developmental and gender‐based differences in children's aggression.

The most notable cross‐country descriptive difference was observed in the act of excluding peers from friendship groups, which was much more common among preschool children in NZ (61.5% vs. 16.1%). This finding is in contrast with the PISA data that shows that 15‐year‐olds in the U.S. and NZ experience comparable percentages of purposeful exclusion (Organisation for Economic Co‐operation and Development, OECD 2023). Several reasons may explain this difference though given the developmental differences in these age groups, these suggestions are somewhat speculative. First, normative beliefs held by adults about relational and physical aggression can influence how such behaviors are managed (Swit et al. 2018). Research with teachers and parents in NZ suggests that relational aggression is perceived as normative, and adults are less likely to intervene in these behaviors (Swit 2021). However, similar patterns have been observed in Canadian samples (Bosacki et al. 2014), suggesting that these perceptions may not be unique to New Zealand. Future research should continue to explore cross‐country comparisons of teachers' and parents' beliefs about specific aggressive behaviors to assess whether these social learning mechanisms contribute to the differences in youth aggression rates. Second, differences in preschool environments may help explain the large variation in exclusionary behaviors observed across countries, and this may provide a more compelling explanation for the observed differences in our study. In NZ, the kindergartens involved in this study consisted of large classrooms with children aged 2–5 years, typically with around 40 children on any given day. The large age range and class sizes may have contributed to higher rates of exclusionary behaviors as children are required to navigate social dynamics and form friendship groups. Further, large classrooms may also make it harder for teachers to observe exclusionary behaviors, which could help explain the low reported rates of such behaviors in the U.S. Future research should explore why large differences in the rates of exclusionary aggression exist between countries.

### 4.2 Relational and Physical Aggression in U.S. Youth

Among youth in the U.S., the most common specific acts of aggression from preschool through first grade were ignoring or stopping talking to peers and pushing and shoving. Both boys and girls demonstrated high percentages of ignoring behaviors, with boys engaging more in this behavior during preschool and kindergarten, while girls showed higher rates by first grade. Boys consistently exhibited higher percentages of pushing and shoving compared to girls across all grade levels. These findings align with the broader literature, which indicates that physical aggression is more common among young children, particularly boys (Côté et al. 2006; Nelson et al. 2022), while gender differences in relational aggression tend to be less pronounced (Nelson et al. 2022; Swit and Slater 2021). The percentages of aggressive acts that require verbal skills, such as telling other kids to beat someone up, and spreading rumors, significantly increased as a function of grade level, a trend seen for both boys and girls. This likely reflects the development and growth of social and verbal abilities across early childhood development (Nelson et al. 2022).

Of all the specific acts of aggression measured, only two relational—spreading rumors and excluding peers from the friendship group—and one physical—telling other kids to beat someone up—showed a significant increase from preschool to first grade. Percentages of all other acts of aggression remained relatively stable across grade levels. Interestingly, certain behaviors such as hitting, pushing, ostracism, ignoring, and spreading rumors, exhibited variability, with lower percentages observed in kindergarten compared to preschool and first grade. These findings are in contrast from previous research, which generally shows a decline in physical aggression and an increase in relational aggression with age (Nelson et al. 2022). However, it's important to note that our study examined cross‐sectional data, comparing children in different grade levels at one point in time. Future longitudinal research should examine the progression of specific acts of aggression as children develop.

### 4.3 Relational and Physical Aggression in NZ Youth

In the NZ sample, findings revealed that reactive functions of aggression were more common than proactive functions across all ages and genders. This pattern in behavior held true for both relational and physical forms of aggression, suggesting that children aged 2–5 years in NZ tend to respond impulsively with aggression rather than engaging in premeditated or goal‐orientated acts of aggression. Small to moderate gender differences were evident in both relational and physical aggression, though not for all acts of aggression. Girls were more likely to engage in specific proactive acts of relational aggression that involved telling a peer that s/he won't be their friend anymore, or saying “you can't come to my birthday party,” to get what they want. Additionally, girls exhibited reactive relational aggression by expressing anger through statements like “I won't be your friend anymore.” In contrast, boys demonstrated higher rates of proactive physical aggression that involved hitting, kicking, and punching to get what they wanted. They also showed reactive physical aggression, often retaliating when hurt or responding by hitting, kicking, and punching when angry. While gender differences in children's use of physical aggression are well established, gender differences in relational aggression are often mixed (Nelson et al. 2022; Swit and Slater 2021). The findings of this study extend previous research by demonstrating that gender differences in children's aggression may be more nuanced than previously realized, revealing significant differences in specific acts of aggression between boys and girls.

Age differences were most pronounced when comparing 4‐year‐olds to 2 year olds, particularly in two reactive relational behaviors: “If other children hurt this child, s/he often keeps them from being in their group of friends” and “When s/he is angry at others, this child will often tell them that s/he won't be their friend anymore.” One proactive relational behavior, in which the child says, “you can't come to my birthday party” to get what they want, was also more common among 4‐year‐olds than to 2‐year‐olds. These specific aggressive acts require some level of theory of mind and verbal articulation to effectively harm peers, which may explain the higher percentages among older children.

While age‐related differences in physical aggression were not significant, percentage patterns aligned with previous findings that physical aggression peaks between 24 and 42 months (Tremblay et al. 2004) and is highest around 3 years of age (Alink et al. 2006). The differential patterns seen in this study with age further support the notion that specific acts of aggression may follow distinct developmental trajectories within this age group (Swit 2019). Notably, in the U.S. sample, children in first grade (aged 5–7 years) demonstrated higher odds of being rated by their teachers as aggressive compared to preschool and kindergarten peers, suggesting a developmental shift in engagement in specific acts of aggression at school entry. Given that the NZ sample included children only up to the age of 5, cross‐country differences may partly reflect developmental timing rather than cultural differences. Future research should use age‐matched cohorts to more precisely distinguish between developmental and cultural influences on specific acts of aggression and continue to explore specific acts of forms and functions of aggression to inform more targeted prevention and intervention.

### 4.4 Limitations and Future Directions

While the current study has many strengths, including the utilization of two samples from different countries and the consideration of gender differences in associations, the current study has several limitations that need to be considered. First, although data from both countries suggest some descriptively similar patterns and indicate that analyses of specific act of aggression are important, and these differences are evident when utilizing different questionnaires, some differences in percentages and gender cannot be directly compared across samples because of the variation in the questionnaires. Further, the U.S. sample did not collect information on specific ages, and the items did not evaluate the function of the aggressive acts. The children in both samples were recruited from specific early childhood settings that may not be representative of broader national populations. This may limit the generalizability of our findings. Additional research examining the same items that assess both form and function of aggression in the same age groups is needed to fully understand these rates across samples. In both samples, early childhood aggression was assessed by teacher report only. Parent and teacher assessment of childhood behavior has been shown to be modest (De Los Reyes and Kazdin 2005), and while parent–teacher agreement has been shown to be higher for childhood externalizing behaviors relative to internalizing behaviors (Rescorla et al. 2014), the results presented here should be viewed with caution. Regarding the ICC results across both studies, variability in teachers' reports of relational aggression items was more pronounced than for physical aggression items. This variability may reflect the covert nature of relational aggression, which is typically more difficult to observe because it involves subtle social interactions between peers. Other potential reasons for this variability include differences in teachers' perceptions of relational and physical aggression, age and gender biases, the contexts in which these behaviors occur (e.g., classroom dynamics), or the children's individual relationships with specific teachers. Additionally, teacher characteristics such as personal experiences, professional training and development, or approaches to aggression may have influenced how these aggressive behaviors are recognized and reported. Where there are large clusters of teachers within schools or kindergartens reporting on individual child aggression, we recommend that researchers use multilevel modeling techniques to account for such clustering effects. Another limitation is that gender differences in items for particular ages were not able to be assessed in the NZ sample, given relatively small sample sizes for particular age groups. Note, however, although statistical gender differences across grade were not examined the U.S. sample, the pattern of gender differences found across grades were very similar, suggesting this may not be crucial. Nonetheless, future research statistically examining gender by age differences in item rates would be of interest. Finally, these studies were cross‐sectional in nature and did not assess individual changes in specific acts of aggression. Research following up the same youth and assessing for changes in rates of specific items of aggression will be an important next step in understanding how these specific behaviors evolve during early childhood to inform developmental models of prevention and intervention. Moreover, future research evaluating what specific acts of aggression are most strongly associated with adjustment outcomes would be helpful in further understanding risk and what behaviors are most important to address in intervention.

## 5 Conclusion

In summary, this study provides important insights into the specific acts of aggression exhibited by young children in both the U.S. and NZ. By moving beyond broad categories to examine specific aggressive acts, our findings highlight both shared and unique patterns in the forms and functions of aggression across cultural and developmental contexts. Notably, physically aggressive acts are common in early childhood in both countries, with boys more frequently engaging in these behaviors, while relationally aggressive acts show more nuanced gender and cultural differences. Overall, our results highlight the value of examining specific acts of aggression to better understand trajectories and inform more targeted approaches to prevention and intervention across early childhood.

## Author Contributions

**Cara S. Swit:** funding acquisition, conceptualization of study, methodology, data collection, formal analysis, writing – original draft, writing – review and editing, project management. **Paula J. Fite:** funding acquisition, conceptualization of study, methodology, data collection, formal analysis, writing – original draft, writing – review and editing. **Seth C. Harty:** conceptualization of study, methodology, writing – original draft, writing – review and editing.

## Conflicts of Interest

The authors declare no conflicts of interest.

## Supporting information

Supporting Material AB.

## Data Availability

The data that support the findings are available from Paula J. Fite (Study 1) and Cara S. Swit (Study 2) upon reasonable request.

## Endnotes

1 Mean level differences were also examined using t‐tests and correlation analyses, and the same pattern of results emerged.
