# Supplementary material for: Zooming in on Early Aggression: A Cross‐Cultural and Developmental Study of Youth in the United States and Aotearoa New Zealand
Source: Aggress Behav. 2025 Jul 7;51(4):e70043. doi: 10.1002/ab.70043 (PMC12235110; doi:10.1002/ab.70043)
Supplement: Supplementary file 1 — Supporting Material AB. [file AB-51-e70043-s001.docx]

**Supplementary Materials**

Supplementary Table 1. ICCs for Teacher Ratings of Relational and Physical Aggression in Study 1

| **Relational Aggression** | ICC _teacher_ |
| --- | --- |
| When mad, gets even by keeping the person from being in their group of friends | .36 |
| When mad at a person, ignores or stops talking to them | .28 |
| Tries to make other kids not like a certain person by spreading rumors about them | .31 |
| **Physical Aggression** |  |
| Hits, kicks, punches others | .04 |
| Pushes and shoves others | .06 |
| Tells other kids beat them up unless to what they say | .41 |

Supplementary Table 2. ICCs for Teachers Ratings of Relational and Physical Aggression in Study 2

| **Relational Aggression** | ICC_teacher_ | |
| --- | --- | --- |
| **Reactive** |  | |
| If other children hurt this child, s/he often keeps them from being in their group of friends (2) | 0.19 | |
| When s/he is angry at others, this child will often tell them that s/he won’t be their friend anymore (9) | 0.06 | |
| When s/he is upset with others, this child will often ignore or stop talking to them (10) | 0.22 | |
| **Proactive** |  | |
| To get what this child wants, s/he often tells others that s/he won’t be their friend anymore (11) | 0.06 | |
| This child often says “you can’t come to my birthday party” to other children to get what s/he wants (13) | 0.11 | |
| To get what this child wants, s/he often will ignore or stop talking to others (14) | 0.29 | |
| **Physical Aggression** | |  |
| **Reactive** | |  |
| When this child is hurt by someone, s/he will often physically fight back (1) | | 0.00 |
| If other children make this child mad, s/he will often physically hurt them (3) | | 0.00 |
| If other children anger this child, s/he will often hit, kick, or punch them (8) | | 0.00 |
| **Proactive** | |  |
| This child often starts physical fights to get what s/he wants (5) | | 0.00 |
| This child often threatens others physically to get what s/he wants (6) | | 0.00 |
| This child often hits, kicks, or punches to get what s/he wants (12) | | 0.00 |

Supplementary Table 3. Binary Logistic Regression for specific acts of aggression across grade levels with preschool as the referent group for Study 1.

| **Aggression Item** |  | **Nagelkerke**  ***R^2^*** | **B** | **Wald χ^2^** | ***p*** | **Odds Ratio** | **95% CI Lower** | **95% CI**  **Upper** |
| --- | --- | --- | --- | --- | --- | --- | --- | --- |
| Hits, kicks, punches others | Kindergarten | .01 | -.32 | .83 | .36 | .73 | .36 | 1.45 |
|  | 1^st^ Grade |  | .06 | .05 | .83 | 1.07 | .60 | 1.90 |
| Pushes and shoves others | Kindergarten | .01 | -.44 | 1.78 | .18 | .65 | .34 | 1.22 |
|  | 1^st^ Grade |  | .05 | .02 | .86 | 1.05 | .62 | 1.79 |
| Tells other kids beat them up unless to what they say | Kindergarten | .08 | 1.08 | 1.62 | .20 | 2.96 | .56 | 15.66 |
|  | 1^st^ Grade |  | 1.97 | 6.82 | .01 | 7.17 | 1.63 | 31.42 |
| When mad, gets even by keeping the person from being in their group of friends | Kindergarten | .08 | -.22 | .25 | .62 | .81 | .35 | 1.87 |
|  | 1^st^ Grade |  | 1.02 | 9.43 | .00 | 2.76 | 1.44 | 5.28 |
| When mad at a person, ignores or stops talking to them | Kindergarten | .01 | -.44 | 1.97 | .16 | .64 | .35 | 1.20 |
|  | 1^st^ Grade |  | -.05 | .03 | .86 | .96 | .56 | 1.62 |
| Tries to make other kids not like a certain person by spreading rumors about them | Kindergarten | .15 | -.37 | .39 | .53 | .69 | .22 | 2.20 |
|  | 1^st^ Grade |  | 1.55 | 14.14 | <.001 | 4.69 | 2.10 | 10.49 |

Note. Referent Grade is preschool.

Supplementary Table 4. Binary Logistic Regression for reactive and proactive relational aggression across age with 4-year-olds as the referent group for Study 2.

| Aggression Item |  | **Nagelkerke *R^2^*** | **B** | **Wald χ^2^** | **p** | **Odds Ratio** | **95% CI Lower** | **Upper** |
| --- | --- | --- | --- | --- | --- | --- | --- | --- |
| Reactive | | | | | | | | |
| If other children hurt this child, s/he often keeps them from being in their group of friends (2) | 2 year olds | .08 | -1.59 | 10.92 | **<.001** | .22 | .09 | .54 |
|  | 3 year olds |  | -.37 | 1.17 | .28 | .69 | .35 | 1.35 |
|  | 5 year olds |  | -.49 | .71 | .40 | .61 | .19 | 1.92 |
| When s/he is angry at others, this child will often tell them that s/he won’t be their friend anymore (9) | 2 year olds | .06 | -1.27 | 7.60 | **.01** | .28 | .12 | .69 |
|  | 3 year olds |  | -.06 | .04 | .85 | .94 | .50 | 1.78 |
|  | 5 year olds |  | .03 | .00 | .96 | 1.03 | .33 | 3.20 |
| When s/he is upset with others, this child will often ignore or stop talking to them (10) | 2 year olds | .02 | -.78 | 3.20 | .07 | .46 | .20 | 1.08 |
|  | 3 year olds |  | -.35 | 1.09 | .30 | .71 | .37 | 1.36 |
|  | 5 year olds |  | -.31 | .28 | .60 | .74 | .24 | 2.30 |
| Proactive | | | | | | | | |
| To get what this child wants, s/he often tells others that s/he won’t be their friend anymore (11) | 2 year olds | .05 | -1.18 | 5.87 | **.02** | .31 | .12 | .80 |
|  | 3 year olds |  | -.05 | .02 | .88 | .95 | .51 | 1.79 |
|  | 5 year olds |  | -.08 | .02 | .89 | .92 | .30 | 2.80 |
| This child often says “you can’t come to my birthday party” to other children to get what s/he wants (13) | 2 year olds | .08 | -1.81 | 7.69 | **.01** | .16 | .05 | .59 |
|  | 3 year olds |  | -.64 | 3.32 | .07 | .53 | .27 | 1.05 |
|  | 5 year olds |  | -.27 | .20 | .66 | .77 | .24 | 2.47 |
| To get what this child wants, s/he often will ignore or stop talking to others (14) | 2 year olds | .03 | -.97 | 3.93 | .05 | .38 | .15 | .99 |
|  | 3 year olds |  | -.10 | .09 | .76 | .91 | .48 | 1.72 |
|  | 5 year olds |  | -.14 | .06 | .81 | .87 | .28 | 2.68 |

*Note:* Bolded values indicate *p* < 0.05.

Supplementary Table 5. Binary Logistic Regression for reactive and proactive physical aggression across age with 4-year-olds as the referent group for Study 2.

| Aggression Item |  | **Nagelkerke *R^2^*** | **B** | **Wald χ^2^** | **p** | **Odds Ratio** | **95% CI Lower** | **Upper** |
| --- | --- | --- | --- | --- | --- | --- | --- | --- |
| Reactive | | | | | | | | |
| When this child is hurt by someone, s/he will often physically fight back (1) | 2 year olds | .04 | -.78 | 3.19 | .07 | .46 | .20 | 1.08 |
|  | 3 year olds |  | .04 | .02 | .89 | 1.05 | .55 | 1.99 |
|  | 5 year olds |  | -.72 | 1.58 | .21 | .49 | .16 | 1.50 |
| If other children make this child mad, s/he will often physically hurt them (3) | 2 year olds | .02 | -.44 | 1.00 | .32 | .64 | .27 | 1.53 |
|  | 3 year olds |  | .11 | .11 | .74 | 1.11 | .59 | 2.10 |
|  | 5 year olds |  | -.54 | .81 | .37 | .59 | .18 | 1.88 |
| If other children anger this child, s/he will often hit, kick, or punch them (8) | 2 year olds | .01 | -.18 | .15 | .70 | .84 | .34 | 2.08 |
|  | 3 year olds |  | .19 | .31 | .58 | 1.21 | .62 | 2.34 |
|  | 5 year olds |  | -.30 | .22 | .64 | .74 | .22 | 2.56 |
| Proactive | | | | | | | | |
| This child often starts physical fights to get what s/he wants (5) | 2 year olds | .01 | .03 | .00 | .97 | 1.03 | .33 | 3.20 |
|  | 3 year olds |  | .40 | .90 | .34 | 1.49 | .66 | 3.37 |
|  | 5 year olds |  | -.20 | .06 | .81 | .82 | .16 | 4.11 |
| This child often threatens others physically to get what s/he wants (6) | 2 year olds | .01 | -.67 | .67 | .41 | .51 | .10 | 2.53 |
|  | 3 year olds |  | -.16 | .10 | .75 | .85 | .31 | 2.34 |
|  | 5 year olds |  | .13 | .03 | .87 | 1.15 | .22 | 5.92 |
| This child often hits, kicks, or punches to get what s/he wants (12) | 2 year olds | .01 | .35 | .43 | .51 | 1.41 | .50 | 3.97 |
|  | 3 year olds |  | .37 | .85 | .36 | 1.45 | .66 | 3.22 |
|  | 5 year olds |  | -.29 | .13 | .72 | .75 | .15 | 3.71 |
